# Participatory-deliberative processes in UK policymaking related to income insecurity as a determinant of health: a scoping review

**DOI:** 10.1332/17442648Y2025D000000053

**Published:** 2025-04-14

**Authors:** Anna Baillie, Gillian Fergie, Mhairi Mackenzie, Kathryn Skivington

**Affiliations:** MRC/CSO Social and Public Health Science Research Unit, School of Health and Wellbeing, University of Glasgow, Glasgow, UK; College of Social Sciences, School of Social and Political Sciences, University of Glasgow, Glasgow, UK; School of Health and Wellbeing, University of Glasgow, Glasgow, UK

**Keywords:** public participation, health inequalities, income, policymaking

## Abstract

**Background:**

Deepening democratic engagement in socio-economic policy domains is of increasing interest to the health inequalities research community. However, there is a recognised gap between theory and the practical application of public participation. Viewing income security as a fundamental determinant of health, this Review investigates how, when and where participatory-deliberative processes (PDPs) were applied in policymaking connected to income, in the UK, from Jan 2007-June 2022.

**Methods:**

The Review applied the PRIMSA-ScR checklist. Searches were conducted in: EconLit, SOC Index, Sociological Abstracts, MedLine; and grey literature sources: BASE database, government, NGO websites for articles related to PDPs in income-related policymaking in the UK, published after 1 January 2007. Articles were synthesised through a conceptual framework combining Whitehead’s typology of actions to tackle health inequalities and Smith’s categorisation of democratic goods.

**Findings:**

The Review found 20 articles relating to 13 PDPs. A majority of PDPs took place in Scottish Government/ Parliament or at Local Authority /NHS Trust level in England and Wales. A variety of types of PDPs were used by policymaking institutions across a range of socio-economic domains, with varying degrees of information provided about participants and policy outcomes.

**Discussion and conclusions:**

Findings demonstrate a multitude of disconnects between participatory rhetoric and reality. There is no evidence of PDPs influencing macro socio-economic policymaking, with participatory decision-making instead dispersed across less empowered, downstream spaces. Democratising socio-economic policy domains requires critical reflection on the fractured nature of participatory policymaking, the locus of decision-making power and how inclusion is realised in participation spaces.

## Background

Social inequalities in health present a persistent and complex policy problem for governments across international and socio-political contexts (Smith et al., 2016). Described as the unfair and avoidable differences in health outcomes across different groups (Miall et al., 2022), recent evidence tells us that, despite a decades-long policy focus and intense, sustained scrutiny from the research community (Smith et al., 2016), progress to reduce health inequalities in the UK has stalled and in some areas begun to widen once more (Walsh et al., 2022, Miall et al., 2022). Theorists argue this is thanks to a failure to address the fundamental drivers of health inequalities related to income, wealth and education (McCartney et al., 2013, Braveman and Gottlieb, 2014, Bambra et al., 2021, McCartney et al., 2023), with a distinct lack of action at the macro level to transform socio-economic policy related to taxation, employment, and social protection systems (Mackenbach, 2011, McCartney et al., 2013, Smith et al., 2016). Indeed, despite the lack of UK progress, there have been long standing and concerted calls from the research community for *progressive economic reforms*, to tackle health inequalities at the roots. And yet these reforms remain elusive, with lack of political appetite (Smith, 2015); complex and plural explanations of health inequalities (Douglas, 2016, Kelly-Irving et al., 2022) and a seemingly immutable policy focus on individual lifestyle choices (Katikireddi et al., 2013) resulting in a ‘policy stalemate’ (Smith et al., 2022) characterised by interventions which routinely fail to address the unequal distribution of power and wealth (Mackenzie et al., 2020).

There is now an emerging cohort of theorists who are calling not just for different *decisions* to be made but for the exploration of different *decision-making processes* (McCartney et al., 2021, Bambra et al., 2021, Smith et al., 2022). Calls for action at the socio-economic policy level are now accompanied with calls for a *democratic rejuvenation* (Bambra et al., 2021), which looks beyond representative voting in elections towards a deepening of democratic practices (McCartney et al., 2021), which ultimately has more public participation in political and policy decision-making at its heart (Bambra et al., 2021). Underpinned by the deliberative notion of pluralism, i.e., the more people and perspectives you have involved in a decision-making process, the better, fairer and more fit for purpose the eventual policy decisions will be (Gutmann and Thompson, 2004, Fishkin, 2018), it has been argued that lay forums may provide a mechanism through which to overcome the health inequalities policy ‘stalemate’ (Smith et al., 2022) and stimulate action to address the fundamental causes. Worldwide there is growing interest in innovative, participatory methods which can be employed by policy stakeholders, in particular deliberative approaches and processes (OECD, 2020), including techniques such as citizen’s juries, panels and assemblies, participatory budgeting and collaborative governance (Fung, 2006, Elstub and Escobar, 2019). These participatory and deliberative decision-making processes are commonly placed under the banner of *democratic innovation*s (Smith, 2009) and it is the exploration of these innovations which health inequalities and democratic theorists alike are calling for (Bambra et al., 2021, Smith et al., 2022, Vlahos et al., 2024). Of course, the idea of public participation is by no means new. The UK has a long history of experiments in and different iterations of participatory functions in governance (Lowndes et al., 2006, Barnes et al., 2007). A wealth of empirical knowledge tells us that citizen participation in policymaking is not a simple endeavour (Dean, 2017, Pallett, 2020). Whilst much lauded, participation in decision-making can be hard to achieve in a way which feels meaningful, and effective, to both members of the public and policymakers (Ganuza and Baiocchi, 2016, Elstub et al., 2019). Indeed, whilst democratising decision-making related to socio-economic policy is an area of growing interest in health inequalities research in the UK (McCartney et al., 2021, Bambra et al., 2021, Smith et al., 2021, McHugh et al., 2023), there is a recognised gap between the democratic theory and practical application at a socio-economic level (Vlahos et al., 2024). This review attempts to bridge that gap. In our current context of post-pandemic, austerity 2.0 and a severe costs of living crisis characterised by increasing financial precarity across the UK (Clark and Wenham, 2022) we use the policy lens of income insecurity to investigate how, when, and where participatory-deliberative processes (Bua, 2017), have been used in income-related policymaking in the UK, from Jan 2007-June 2022. Income insecurity was chosen as the policy lens as it provides a broad term which links into different aspects of socio-economic decision-making, such as social security, employment and poverty, which have been identified as crucial domains for action to tackle health inequalities (McCartney et al., 2013, Smith et al., 2016, Bambra et al., 2021). Similarly, the term ‘participatory-deliberative processes’ (PDPs) (Bua, 2017) is purposefully broad to reflect the ‘range of institutional possibilities for public participation’ (Fung, 2006) as well as the increasing application of, or focus on, deliberation as a participatory function (Gutmann and Thompson, 2004, OECD, 2020). We reviewed and synthesised empirical and real-world evidence, with a particular focus on PDPs which were connected to real-world policy settings, to understand and articulate any lessons which might inform a *democratic rejuvenation* of policymaking to tackle the socio-economic drivers of health inequalities.

## Methods

This review follows the framework for scoping studies developed by Arksey and O’Malley (2005), which sets out steps relating to: identifying research questions and relevant studies; selecting studies; synthesising and interpreting data; collating, summarising and reporting findings. We also drew upon Levac et al. (2010), who drew on their experience of applying the framework to three scoping studies, to enhance and clarify each step, advancing the framework further. It is structured in accordance with the Joanna Briggs Institute’s guidance for conducting systematic scoping reviews (Peters et al., 2015) and applied the Preferred Reporting Items for Systematic Review and Meta-Analysis Extension for Scoping Reviews (PRIMSA-ScR) checklist (Tricco et al., 2018). It builds on previous work to understand the usage of PDPs, particularly in a healthcare setting (Street et al., 2014, Degeling et al., 2015, Baumann and Brütt, 2021), as well as a prior rapid review of community engagement initiatives employed to tackle the social determinants of health (Popay et al., 2007). Previous reviews have tended to focus on either a specific method, such as a citizen’s jury (Street et al., 2014); decision-making in health care settings alone (Street et al., 2014, Baumann and Brütt, 2021); the type of public participating (Degeling et al., 2015), or multiple approaches over vast policy domains (Popay et al., 2007). This scoping review considers both peer-reviewed and grey literature related to participatory-deliberative processes (PDPs) occurring in policy domains connected to income insecurity, with the following aims:

- Examine how, when and where PDPs have been applied in policymaking related to income insecurity in the UK.
- Understand how these processes influence policy outcomes and at what level, using a combined analytical framework of Whitehead’s typology of actions to tackle social inequalities in health (2007) and Smith’s categorisation of democratic goods (2009).

### Searches

The search terms and strategy were informed by Peters et al.’s Population, Concept, Context (PCC) framework (2017) as well as the prior rapid review of community engagement related to tackling the social determinants of health (Popay et al., 2007). Both were developed iteratively, with input from all authors. The previous international review of community engagement initiatives found the literature related to community involvement to be vast and varied (Popay et al., 2007), meaning clear selection criteria was necessary to find the most pertinent studies. Although the focus was different for that review, there is overlap with the population and concept elements of this review. For this reason, studies were selected from 2007 onwards to avoid any duplication and clearly differentiate this study in its own right. In addition, from the financial crash in 2008, the UK witnessed significant reforms to socio-economic policy, in the form of austerity (Bartley and Blane, 2016) so there is wisdom in considering this distinct period in UK policymaking. Furthermore, the decision to focus on UK only is largely pragmatic to allow for feasibility in terms of the number of potential studies to review as well as simplifying the policymaking context to UK-wide rather than international. Key search terms included ‘population’ terms, for example “lived experience”, “low-income”, “lay”; ‘concept’ terms, for example “participation” “decision making”, and “democratic innovation’; and ‘context’ terms, for example “policy making”, economic policy’, “income insecurity’, “poverty”. Searches were conducted in peer-reviewed databases: EconLit, SOC Index, Sociological Abstracts, and MedLine, as well as grey literature sources, including BASE database, government, third sector, and thinktank websites for articles related PDPs in income-related policymaking from Jan 2007-June 2022. The full search strategy is available in the Supplementary File (available at https://doi.org/10.6084/m9.figshare.28623641).

### Inclusion strategy

Initial searches returned 14,413 articles; 21 duplicates and 13,727 non-UK articles were removed. The lead author was the primary reviewer and conducted the initial screening for UK-based examples. The Title and abstract (N=657) and Full text review (N=180) stages were completed with support from the research team, with around 10% of articles double-screened at each stage to ensure consistency in the application of the inclusion strategy, detailed in Table 1.

**Figure 1:**
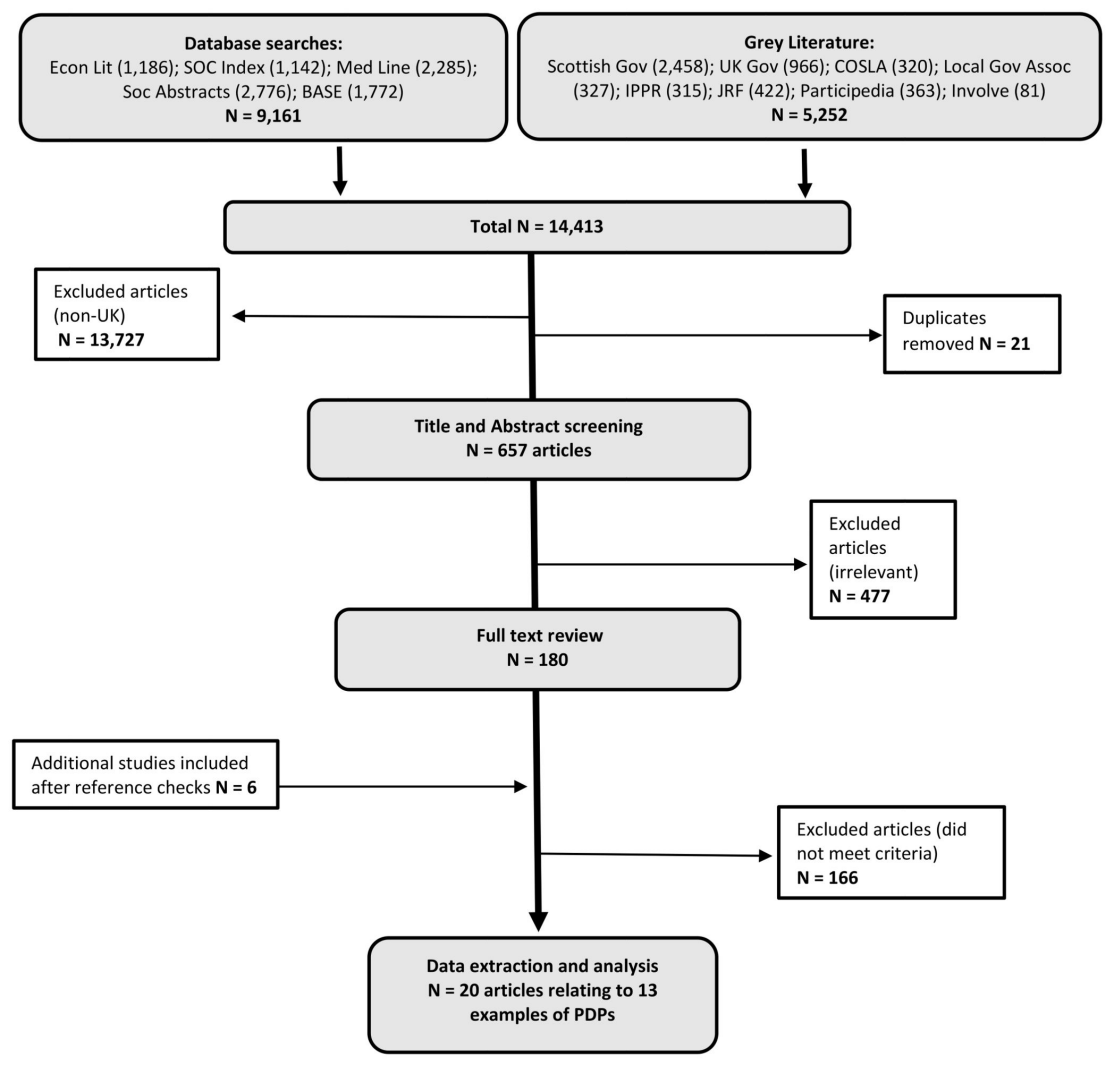
Searches A flow chart showing the number of articles returned from the searches, and the process of exclusion. There were 9,161 articles returned from database searches and 5,252 from grey literature searches. The total number of articles retrieved, once 21 duplicates were removed and 13,727 non-UK articles were removed, was 657. At title and abstract screening 477 articles were removed as they were irrelevant. The full texts of 180 articles were reviewed, plus a further 6 that were retrieved after reference checks. 166 articles were excluded as they did not meet inclusion criteria. Twenty articles were taken forward to the data extraction and analysis stage, which related to 13 examples of PDPs.

Articles were excluded if they did not contain enough information on the PDP, if the policy domain being explored was not sufficiently detailed, or if there was no discernible to connection to ‘income-related policy’, for example healthcare policymaking. Similarly, informed by Fung’s Democracy Cube (Fung, 2006), processes which were focused solely on the participation of expert administrators, elected representatives or professional stakeholders were excluded. This is because this review is primarily interested in the processes and opportunities for *citizens* to participate in, not technocrats. The research team met regularly throughout the screening process to discuss the included articles. Any disagreements were reassessed and discussed as a group to reach final decisions on inclusion. A total of 20 articles relating to 13 examples of PDPs were included for data extraction and analysis, as detailed in Figure 1. For simplicity, the Review considered each PDP (N=13) as the unit of analysis, even where there were a cluster of documents relating to one process.

### Conceptual analysis

The analysis of the articles in this review is two-fold, relating to both policy domain and the application of PDPs. The literature was synthesised through Whitehead’s typology of actions to tackle social inequalities in health (2007), a theoretical tool for understanding the various, and often common, loci for policy action, consisting of four categories: Strengthening individuals, Strengthening communities, Improving living and working conditions, and Promoting healthy macro policies. Both the *policy ambitions* of the PDPs, i.e., the policy problems and domains they were aiming to influence or solve, as well as their *participatory outcomes*, i.e., the type of decisions citizens were involved in or the recommendations they produced, were mapped onto Whitehead’s typology, providing helpful detail on the *types* of policy problems and *levels* of decision-making presented via the PDPs.

The second aspect of analysis considers the PDPs through a democratic theory lens to evaluate how the processes operate and unfold in income-related policy settings. Smith’s analytic framework of *democratic goods* (2009) provides a helpful tool in this context. In its fullest version Smith’s framework consists of four ‘explicit’ democratic goods: *inclusiveness, transparency, popular control*, and *considered judgement* as well as two institutional goods: *efficiency* and *transferability* (Smith, 2009). Whilst each have merits in understanding institutionalised participatory mechanisms, it is not possible to understand all six with the data available in this scoping review, given the, at times limited, reporting on each PDP covered in the Scoping Review. Considered judgement, i.e., whether the decisions of citizens are based on ‘raw preferences… rather than on an informed and reflective assessment of the matter in hand’ (Smith, 2009) in particular is challenging. The framework is therefore simplified to three democratic goods, which overlap, and allow investigation of each process in the context of the data available:

- *Inclusiveness*: Who is participating?
- *Transparency*: How clear is the process design and decision-making within it?
- *Popular control*: What evidence is there of citizen influence on policy as a result of the PDP?

This combined analytic framework illuminates the PDPs in terms of the policy context, process design and impact, locating the review firmly in contemporary conversations considering the utility of deepening public participation in policymaking to tackle the socio-economic drivers of health inequalities.

## Findings

The findings are presented below in two parts. Firstly, the characteristics of the synthesised literature are discussed, with reference to the source and location of articles, as well as the policy problems explored and the participating citizens. Then three key areas of disconnect across the PDPs are highlighted: curated citizen input; wide remits with little accountability; and incongruous conclusions/recommendations.

### Characteristics of PDPs

#### Type of literature

There are common characteristics across the literature included in this review. The articles detail, in varying degrees, the length of process, participants involved, themes from discussions and the resulting participatory outcomes, which are, for the most part, in the form of a stand-alone suite of citizen recommendations. We sought real world examples and as such the majority of PDPs are detailed via grey literature sources in the form of a final report (N=11), often produced by an external consultant funded to deliver the PDP. Much has been written about the emerging ‘participation industry’ and the tensions this can produce for public engagement professionals who serve organisational as well as democratic interests (Behrer and Lee, 2019). It is perhaps for this reason the reports tend to be descriptive in nature rather than applying a critical lens to the PDPs and their outcomes. This means that observations cannot be made on how well these articles represent the process as it was experienced by citizens or on the level of synthesis employed by the consultants to produce the final report. However, as records of the PDPs, the reports provide a helpful snapshot of participatory activity in socio-economic domains, which, when observed through the conceptual framework of this review, reveal clear themes, related to both the data that are included and some notable silences. Full detail of the included articles can be found in Table 2.

#### Institutional spread & type of process

The 13 examples vary in terms of location and type of process. Publications from Scotland were in the majority, with all of the included studies from that country convened or funded by Scottish Government or Scottish Parliament (N=6), whilst the next biggest cohort occurs at Local Authority and/or NHS Trust level across England and Wales (N=4). The returns from UK Government totalled two. Despite the relatively small number of examples found in the review, there were a variety of types of PDPs used by institutions. Citizens’ juries and assemblies were the most common, representing over half of the processes analysed (N=7), with other techniques such as workshops (N=3), collaborative governance, where citizens work alongside or with institutional representatives, (N=2) and participatory online forums (N=1) used less frequently. As detailed above, there was relative consistency in the type of outputs from each process, with almost all producing a suite of stand-alone recommendations or elicited suggestions from citizens for the consideration of the convening institution (N=12).

#### Who participated?

Inclusion in terms of presence is of concern, in some respect, to the majority of examples included in this review. However, it varies, sometimes significantly, in the degree of attention it is given. Some of the examples give detailed breakdowns of *who* is involved in the process, often related to socio-economic demographics such as age, gender, ethnicity and geographical residence (Scottish Parliament, 2021) with some including additional information on social grade and working status (Scott, 2017), educational qualifications and voting history (Involve, 2019) or in two cases, detailed information on protected characteristics (Scottish Government, 2020a, Involve, 2021). However, most provide relatively scant detail on the citizens participating, stating that members consisted of a cross-section of society or had ‘lived expertise’ of a particular issue or place, but giving no, or very little, further detail about the participating citizens (N=8).

There is across the review an incongruous approach to inclusiveness, which is largely characterised by missing information. Even where detailed breakdowns of participating citizens are provided, it is rare for them to be accompanied by rationale as to why these characteristics were important for each process or the particular policy problems being explored, meaning it is difficult to make a comprehensive assessment on how inclusive the PDPs were, a challenge encountered in previous reviews (Street et al., 2014). Democratic theorists have long been concerned with the issue of inclusion, and equality, in participatory spaces (Young, 2000), often recognising that democratic processes do not organically eliminate structural inequalities (Fraser, 1990, Smith, 2009) and indeed have the potential to further entrench deeply unequal power structures (Ganuza and Baiocchi, 2016, Rolfe, 2018). This point is particularly salient in the context of social inequalities. Political participation, of any kind, is unequal (Lijphart, 1997), with people at the lower socio-economic end of the social gradient less likely to participate and therefore less likely to yield any influence (Dalton, 2017). Important questions like ‘who is participating, and why?’ are left largely, or at least partially, unanswered by the literature in this review, which raises valid questions about how inclusion is conceptualised, and importantly evidenced, in PDPs. Similarly, representativeness in terms of scale is largely ignored by the examples included here, all of which constitute small groups of citizens participating, raising questions about the democratic validity of any potential decision-making which impacts on population-wide policymaking. Indeed, in order to fully realise the potential of any democratic rejuvenation as a means of redistributing power to address social inequalities, and the health differences that flow from these, these questions need to be explicitly considered by researchers and advocates, as well as those convening PDPs, to mitigate potential risks of entrenching existing inequalities.

#### Types & levels of policy problems

The cross-sectoral nature of income-related policy is reflected in the scope of policy domains and problems tackled via the PDPs. Income insecurity is recognised explicitly by three studies included in the review, with the rest connected via inter-linked policy areas, detailed in Table 3.

Although the examples of PDPs included in the Review are small in number, the spectrum of topic areas revealed above demonstrate that participatory activity is indeed already taking place across a range of interconnected socio-economic domains in the UK potentially good news for those wishing to deepen democratic engagement in these areas.

## Discussion

### Confronting disconnects

When mapped onto Whitehead’s typology of actions to tackle social inequalities in health (2007), all of the policy problems and domains which the PDPs were aiming to influence or solve fell, at least in part, into the upper echelons: Improving Living & Working Conditions or Promoting Macro Healthy Policies. However, on closer analysis, this upstream policy focus, (i.e., those that focus on prevention by addressing the root causes of a social or health-related issue rather than acting downstream to target the symptoms of it, McKinlay, 1979)is by no means static across the PDPs, and becomes fractured, dispersed or undermined when each process is considered through the conceptual framework. Indeed, this Review finds there are multiple ways in which the PDPs disconnect from their policy ambitions to influence income-related policymaking. These disconnects can largely be grouped according to the following three themes:

1.1 Upstream policy problem, downstream citizen input
1.2 Big remits, little accountability
1.3 Citizen recommendations: a confused smorgasbord

### 1.1 Upstream policy problem, downstream citizen input

Whilst the PDPs included in this review often have the stated *intention* of influencing upstream socio-economic policy domains, when the participatory outcomes of the processes are considered, citizen input is often firmly bound or curated by the convening institution. This is illustrated in the Experience Panels convened by Social Security Scotland, which cite significant influence over policy development, promising that panel members will ‘inform all the key decisions across the design of social security (Scottish Government, 2019). However, the policy aspects which the panel members are asked to co-design are less to do with the substantive policy detail, such as the *rate* of benefits, and more to do with the style and wording of award letters, branding, and application process design.

We see a similar curation of citizen input in other examples. In two of the peer-reviewed articles describing an urban regeneration initiative to tackle poverty and deprivation, the relegation of citizen input is severely critiqued (Dicks, 2014, Adamson, 2010). In this case, participation is fixed at the individual/ community level, with citizen input constantly mediated by officials in cross-sectoral partnership meetings (Adamson, 2010) and often limited to participation in pre-determined, skills-based groups, essentially precluding any exploration of the upstream drivers of poverty (Dicks, 2014). This slip downwards is witnessed again in a citizens assembly convened to tackle health inequalities which instructed citizens to produce recommendations at two levels: how services can help and what individuals and communities can do (Kaleidescope, 2020), revealing a tacit proscription of deliberations related to systemic change, meaning citizen discussions were purposefully directed towards downstream action from the very beginning of the process. We find, in these examples, a general disconnect between the stated policy problem the PDP is convened to solve and the actual policy window available for citizens to input into, with a tendency to shift away from upstream, socio-economic policy discussions towards action at the *strengthening individual and community* levels (Whitehead, 2007).

### 1.2 Big remits, little accountability

We meet a related, although different, disconnect in other PDPs around the remit/intention of the process. In some cases, citizen contributions and deliberations have evidently *not* been limited to downstream areas, with citizens producing suites of wide-ranging and multi-level policy recommendations. When assessing levels of *popular control*, i.e., citizen influence, this appears to present a different challenge: impossibly large remits. The accompanying research for one example, which posed a series of open questions, came to the conclusion that the breadth of remit might hinder its capacity to influence policy (Elstub et al., 2022). This PDP produced sixty citizen recommendations in eight, cross-cutting policy areas (Scottish Government, 2021), meaning the proposals had no specific policy home and therefore no obvious mechanism of accountability.

Whilst comprehensive assessments of the policy legacies of the PDPs included here is outwith the scope of this review, lack of *built-in* accountability is a key theme. There are a small number of PDPs (N=4) which detail their eventual policy impact. However, for the most part, the impact they detail is negligible (N=3), or difficult to attribute to the participatory process itself (N=1). Indeed, explicit policy change as a direct result of PDPs appears illusive. Whilst some examples name the steering or strategy group the recommendations will be considered by (Scott, 2017, Kaleidescope, 2020, Involve, 2021), this is rarely accompanied with a commitment and timeline to respond and only one details a formal response to the citizens (Scottish Government, 2021). In fact, there are four which do not detail, in any way, the planned next steps or means through which the PDP will connect to the specific policy departments identified for change in their recommendations (Involve, 2019, Cowan, 2020, The Poverty Truth Commission, 2020, Scottish Parliament, 2021). This is a loud silence in a review which purposefully sought *institutionalised* participatory mechanisms, supposedly connected, by design, to policymaking spaces. It suggests that citizen deliberations are sometimes wholly segregated from decision-making, with no visible route to eventual policy influence

### 1.3 Citizen recommendations: a confused smorgasbord

Our third disconnect relates to the framing of policy problems throughout the PDPs. Ranging from seventeen up to sixty, the suites of policy proposals which emerge from the included examples can be at once wide-ranging and, at times, contradictory. This is evident in the three citizens’ juries and assemblies convened to answer a question related to health inequalities (Byrant and Beddow, 2017, Kaleidescope, 2020, Involve, 2021). The recommendations from all three examples present a complicated web of policy recommendations and incongruent assumptions about the root causes of the problem. This is illustrated in one example’s dual conceptualisation of unemployment, its drivers and potential solutions (Byrant and Beddow, 2017). Of the thirty citizen recommendations produced, two relate to unemployment as a determinant of health, however they provide different narratives for the locus of the problem, and as such the recommended policy action, as detailed in Table 4.

It reveals a familiar cross-cutting tension about the genesis of issues like unemployment and health inequalities: individual capacity versus upstream action (Katikireddi et al., 2013, Mead et al., 2022). Recommendations which recognise macro socio-economic determinants often sit alongside those which distil health inequalities through lifestyle-based policy solutions. These ‘disrupted narratives’ (Mackenzie et al., 2020) have been found in other studies, which, in turn, suggest such plural and paradoxical perspectives present opportunities for debate and deliberation (Mackenzie et al., 2020). However, across the review, it is unclear how the inconsistencies and contradictions evident in the suites of recommendations were deliberated and reconciled by citizens through the process, with many examples providing records of the voting or prioritisation exercise that led to the final recommendations rather than detailed information on citizen discussions.

Indeed, *how* the policy problems, and any associated theories for their existence, were framed in the participatory processes is relatively unclear. This is a common theme across the PDPs where ’expert’ contributions are routinely cited but rarely detailed, with two notable exceptions (Citizen's Assembly Scotland, 2020, Scottish Parliament, 2021). Given the policy problems being discussed across this review are complex and cross-sectoral, there is a notable lack of transparency around how evidence, theory and expertise was brought in to support citizens to unpick these often-complicated problems. In the context of deliberative democracy, exposure to a range of ideas is seen as crucial in terms of legitimacy and sound decision-making (Fishkin, 2018). Without detail given on the nature and content of evidence provided by ‘experts’ as well as the rationale for requesting their input, it is difficult to know if this plurality of perspectives was achieved. This perhaps goes some way to explaining the confused smorgasbord of citizen recommendations produced as a result, which can appear incoherent and, at times, disconnected from contemporary theory and weight of evidence.

The three disconnects witnessed across the review demonstrate how complex and multi-layered designing a PDP can be in real-world policy settings. Despite relatively ambitious *intentions* to influence income-related policy, citizen participation appears confined to downstream, process-based policy windows or segregated from policy decision-making altogether, routinely characterised by a lack of built-in accountability. This is further exacerbated by an opaque use of evidence and expertise which leaves the door open to disrupted narratives, which appear unexplored and the opportunity for in-depth deliberation potentially lost.

### Strengths and Limitations

This review contributes to contemporary discussions exploring participatory-deliberative fora in the context of health inequalities (McCartney et al., 2021, Smith et al., 2022, McHugh et al., 2023). It offers a practical perspective on the current usage of PDPs in socio-economic domains. Whilst the review was comprehensive, our focus on real-world examples means that searches took place across a variety of platforms, each with their own search engines. It was outwith the scope of this review to develop institution-specific search strategies for each platform; therefore, it is likely that examples were missed thanks to being categorised differently from the search terms or subsumed in broader policy areas. We also note that the articles included in the review are unable to tell the whole story of each process, meaning the analysis is as much focused on the data included as what is missing. This limitation is particularly pertinent in the context of how evidence and expertise inform the decision-making of citizens within participatory spaces, i.e., Smith’s democratic good of ‘considered judgement’ (2009). It was not possible to fully explore with the data available in this review but clearly merits further empirical investigation. Further research is warranted to explore how, who and what expertise is brought into PDPs as well as how this then interacts with citizen perspectives and the identified tensions between support for upstream and downstream interventions to tackle inequalities. In addition, we recognise this review does not include examples from research and third sector settings, which also offer a rich source of learning related to participatory policymaking.

## Conclusion

This review provides a sense check for any democratic rejuvenation to tackle social inequalities in health. Whilst there *is* public participation taking place in socio-economic policy domains, and in particular related to tackling health inequalities and poverty, this review finds little evidence that PDPs yield any influence on typically closed, income-related policymaking (Oswald et al., 2018), for example, substantive decision-making related to social security, employment or public spending. We find there are a multitude of disconnects between the PDPs, the problem being discussed and suggested policy solutions. These disconnects suggest that PDPs do not always present ‘constructive discursive spaces’ (Smith et al., 2022) and that further empirical exploration is required to deepen our understanding of how participation unfolds in socio-economic settings. A particular focus is required on process design including how policy problems and their potential solutions are framed within the space as well as the role of contemporary evidence and expertise, a point reflected in wider contemporary studies which consider how public participation and policymaking interact (Smith-Merry, 2020, Pallett, 2020, Hill O’Connor et al., 2023). Indeed, understanding the distinct policy impact of public participation processes is a contemporary concern for theorists (Bussu et al., 2022b, Russell, 2017, Demski and Capstick, 2022), who are increasingly focused on understanding how PDPs interact with the policymaking systems in which they are taking place, i.e., their ‘embeddedness’ within decision-making (Bussu et al., 2022a). This review suggests there is work to be done to understand the role(s) which PDPs actually play within policymaking settings. Similarly, asking questions related to inclusion of the PDPs, such as ‘who is participating, and why?’ appear important in exploring whether experiences of inequality and marginalisation are potentially being replicated within participatory processes. Finally, critical reflection is also required on the locus of decision-making power, in particular, the actual policy window open for citizen input, and, crucially, whether participatory rhetoric matches the reality.

## Supplementary Material

Supplementary File

## Figures and Tables

**Table 1:** Inclusion strategy

Inclusion criteria for selecting articles
UK Based, i.e., the PDP took place in the context of policymaking within the UK.
Published between Jan 2007-June 2022
Details a PDP which involved citizen interaction and discussion
Examples of direct & hybrid democracy (Weakley and Escobar, 2018), i.e., directly involving citizens rather than solely professional stakeholders
Details an ‘institutionalised mechanism’ (Smith, 2009) which was funded and / or convened by a policymaking institution
Relates to ‘income-related policy’, i.e., tax, spending, debt, social security, poverty or broad governance arrangements of institutions which impact on how income-related policy decisions are made

**Table 2:** Data extraction

Reference	Type of document	Type of PDP	Who participated?	Policy ambitions: aims and objectives of PDP	Evidence and expertise	Participatory outcomes
***1. Spending Review 2010: Jury’s Verdict*** (Mellor and Cooke, 2010)	Grey literature Final report produced by external consultant	Citizens’Jury	24 x citizens who were ‘broadly representative of the population’, i.e., mini-public	Report states the jury was convened 'to help inform the Coalition Government’s thinking by providing insight into citizens’ attitudes and views on the deficit, and to understand the criteria important to citizens that government should use when selecting where and how to make cuts in public spending^1^. The report also details the follow up meeting to review specific policy areas of education reform and charging for public services.	Representatives brought in from UK Government and various thinktanks (including the facilitating organisation). No information provided on format and content of evidence and expertise.	- Produced a suite of Jury ‘findings’ and verdicts in support of the spending review, educational reform and charging for public services.
***2. Community empowerment: Identifying the barriers to “purposeful” citizen participation*** (Adamson, 2010) ***Participatory Community Regeneration: A Discussion of Risks, Accountability and Crisis in Devolved Wales*** (Dicks, 2014)	Peer-reviewed Qualitative evidence analyses	Collaborative governance	Residents from ‘disadvantaged’ areas in Wales (No info provided on numbers)	To provide opportunities for residents to influence state-funded regeneration process in disadvantaged communities in Wales. Aimed at ‘programme-bending^1^ where community input influenced how local services were delivered and the development of ‘priorities for action in each communit/	No information provided on how or if evidence and expertise was brought into the participatory space.	- Regular citizen representation in planning partnership board meetings and pre-determined thematic sub-groups
**3. *Deliberative Manoeuvres in the Digital Darkness: e-Democracy Policy in the UK*** (Moss and Coleman, 2014)	Peer-reviewed Qualitative survey & evaluation	Participatory online forum	Crowdsourcing, no exact details	An online platform *(The Spending Challenge)* aimed at involving the public in identifying ways ‘to reduce the deficit by cutting public spending in a way that is fair and responsible^1^ Largely aimed at online deliberation and generation of ideas to cut public spending at the UK Government level.	No information provided on how or if evidence and expertise was brought into the participatory space.	- High level of participation: over 100,000 suggestions made or interacted with on the forum
**4. *Public Perspectives of the Collaborative Economy*** (Scott, 2017)	Grey literature Final report produced by external consultant	Deliberative workshops	50 x citizens of Glasgow & Edinburgh recruited to reflect a ‘cross section of the population within these cities’, i.e., a mini-public	To ‘compliment and inform the work of the Expert Advisory Panel on the Collaborative Economy’ convened to support policy development at Scottish Government level.	Participants provided with a variety of information related to the Collaborative Economy via the facilitating organisation as well as one external presentation from third sector organisation. Some information provided on the format and content of evidence and expertise.	- Produced a suite of ‘Key Messages’ for the Expert Advisory Panel on the Collaborative Economy
**5. *Blackpool Far North Health and Well Being Inquiry*** (Byrant and Beddow, 2017)	Grey literature Final report produced by external consultant	Citizens’ Jury	26 x citizens of Blackpool North, aiming for a ‘diverse group of local residents’, i.e., a mini-public	To develop recommendations for the Local Authority & NHS Clinical Commissioning Group in answer to the following question: “For people living in Blackpool Far North what are the main things that affect people’s health and wellbeing and what can be done about them?” In addition, a top-level aim for the inquiry was to support a move away from medical-based interpretations of health towards recognition of its social determinants.	Representatives brought in from local services and one national think tank. Information provided on the format of evidence and expertise, but not the content.	- Produced a suite of 30 recommendations which were ranked according to priority.
***6. Social Security Lived Experience Panels Annual Reports*** (Scottish Government, 2018b, Scottish Government, 2019, Scottish Government, 2020a, Scottish Government, 2020b, Scottish Government, 2022)	Grey Literature 4 x Annual reports 1 x report on panel members’ demographi cs produced by convening institution	Collaborative Governance	Circa 3000 Scottish citizens with lived experience of accessing the UK benefits system	Lived experience panels convened across a variety of characteristics and experiences of benefits to ‘inform all the key decisions across the design of social security* in Scotland.	Information provided on the topics discussed, but no detail on how or if evidence and expertise was brought into the participatory space.	- Co-produced ‘Our Charter’ to guide the development of the Social Security Agency and its founding principles - Citizen input on decisions related to: ○ Style and wording of award letters ○ Branding of the new agency ○ Application process design
**7. *Citizen’s Assembly of Wales - Final Report*** (Involve, 2019)	Grey Literature Final report produced by external consultant	Citizens’ Assembly	60 x citizens of Wales recruited via a ‘civic lottery*, i.e., a mini-public	The Citizens’ Assembly was convened to answer the following question: ‘How can people in Wales shape their future through the work of the National Assembly for Wales?’ It focused on the ways in which citizens might inform on the work of the Welsh Assembly, with a particular focus on how citizens might be involved in economic decisions.	Representatives from academia and think tanks brought into the participatory space. Some information provided on the format and content of the evidence and expertise.	- Produced a series of results from voting/ ranking exercises, alongside rational and notes from deliberations on pros and cons
8. ***A* *Fairer Scotland? Informing the Scottish Government on the real impact of poverty through lived experience*** (The Poverty Truth Commission, 2020)	Grey Literature Final report produced by external consultant	Deliberative working groups	People with lived experience of poverty connected to working groups across Scotland (No info provided on numbers)	To provide Scottish Government with detail on the work and ‘impact^1^ of the Poverty Truth Commissions across Scotland and ‘inform a new set of actions towards a fairer Scotland’.	No information provided on how or if evidence and expertise was brought into the participatory space.	- Produced a suite of themes from the working groups as well as a number of ‘calls for action’
***9. Our Lives, Our Solutions: The Annual Report of the Get Heard Scotland Project*** (Cowan, 2020)	Grey Literature Final report produced by external consultant	Deliberative workshops and events	Circa 200 people with lived experience of poverty from across Scotland	To ‘help people on low incomes get their voices heard on the policies and decisions that most impact their lives and communities’ with a particular view to influencing Every Child Every Chance, Scottish Government’s Tackling Child Poverty Delivery Plan 2018-2022 (Scottish Government, 2018a).	No information provided on how or if evidence and expertise was brought into the participatory space. However, final recommendations ‘shaped by’ an Evidence Analysis Group populated by community activists with lived experience of poverty, but no further detail provided.	- Produced a suite of 60 policy solutions and detail on key themes from the discussions
**10. *Camden Health and Care Citizen’s Assembly-Final Report 2020*** (Kaleidoscope, 2020)	Grey literature Final report produced by external consultant	Citizens’ Assembly	Residents of Camden. (No info provided on numbers)	To inform the Camden’s Joint Health and Wellbeing Strategy and build on the previous work of the Neighbourhood Assembly across three priority areas: - Health inequalities, - Staying healthy and well - Local service provision	Representatives brought in from local services and national organisations. Some information provided on the content of evidence and expertise, but no detail provided on the format.	- Produced a suite of ‘expectations’ across the three priority areas aimed at: ○ How services can help ○ How the community and individuals can help
**11. *Doing Politics Differently: The report of the Citizen’s Assembly of Scotland*** (Citizen’s Assembly Scotland, 2020) ***Scottish Government Response to Doing Politics Differently - The Report of the Citizens’ Assembly of Scotland*** (Scottish Government, 2021)	Grey literature 1 x final report produced by the Citizens’ Assembly; 1 x report produced by the convening institution	Citizens’ Assembly	104 x citizens ‘broadly representative cross-section of Scotland’, i.e., a mini-public	Citizen’s assembly was convened to answer the following questions: What kind of country are we seeking to build? How best can we overcome the challenges Scotland and the world face in the 21st century, including those arising from Brexit? What further work should be carried out to give us the information we need to make informed choices about the future of the country? The aim was to develop a set of actions to be taken forward by Scottish Government.	Wide range of representatives brought in from across academic, political and third sector settings. Full detail provided on both the format and content of the evidence and expertise brought into the participatory space.	- Produced a shared ‘Vision’ and a suite of 60 cross-sectoral recommendations. - Scottish Government published an official response to the recommendations of the assembly.
***Research Report on the Citizens’ Assembly of Scotland*** (Elstub et al., 2022)	1 x research report produced by collaborativ e research group and convening institution					
**12. Scottish Parliament Citizens’ Panel on COVID-19** (Scottish Parliament, 2021)	Grey literature Final report produced by the convening institution	Citizens’Jury	20 x citizens who were ‘randomly selected and stratified’, i.e., a mini-public	The panel was convened to answer the following question: ‘What priorities should shape the Scottish Government’s approach to COVID-19’s restrictions and strategy in 2021?” In response to the four harms of COVID-19: Direct & Indirect health impacts, Social and Economic	Range of representatives from across academic, political, third sector and media settings, with the majority of input coming from academia. Full detail provided on both the format and content of the evidence and expertise brought into the participatory space.	- Produced a suite of 26 recommendations across the four harms
***13. Bristol Citizen’s Assembly: how do we recover from COVID-19 and create a better future for all in Bristol?*** (Involve, 2021)	Grey literature Final report produced by external consultant and convening institution	Citizens’ Assembly	60 x citizens ‘reflective of Bristol’s local diversity’, i.e., a mini-public	Convened to answer the following overarching question: “How do we recover from COVID-19 and create a better future for all in Bristol?” With ‘deep dives’ into the following topics: - Climate Change - Transport - Health Inequalities In order to feed into the One City Economy Board to influence the COVID-19 recovery strategy as well as into Bristol City Council’s Cabinet.	Detail provided on membership of an Advisory Panel who suooorted ‘comprehensive, accurate and balanced’ evidence provision for the Assembly. In addition, external speakers categorised into: - Impartial specialists - Advocates - Experts by Experience However, no further detail provided on format and content of evidence and expertise brought into the participatory space.	- Produced a suite of 17 recommendations, with accompanying detail and rational and how it can be implemented

**Table 3:** Policy domains

Policy domain	No. of PDPs	Specific topics and policy issues the PDP was aiming to solve or influence	References
Poverty	3	Tackling poverty & deprivation, income & food insecurity, income inequality, social security & employment	(Cowan, 2020, The Poverty Truth Commission, 2020, Dicks, 2014, Adamson, 2010)
Public Health	3	Tackling health inequalities, with foci on employment, income, access to discretionary funds and food insecurity	(Kaleidescope, 2020, Byrant and Beddow, 2017, Involve, 2021)
Public Spending	2	Social security, poverty & access to public services with a focus on cuts to funding	(Mellor and Cooke, 2010, Moss and Coleman, 2014)
COVID-19	1	The four harms of COVID-19: direct & indirect health impacts as well as impacts at the economic & societal level	(Scottish Parliament, 2021)
Economy	1	The ‘Collaborative Economy’, including, pay, conditions and security for workers involved in the gig economy.	(Scott, 2017)
Social Security	1	The development of income-based social security entitlements.	(Scottish Government, 2018b, Scottish Government, 2019, Scottish Government, 2020b, Scottish Government, 2020a, Scottish Government, 2022)
Democracy and Participation	1	Involving citizens in decision-making around financial/ resource allocation and general budgeting.	(Involve, 2019)
Multiple & overarching	1	Very broad: Incomes and poverty, tax and economy, young people, sustainability, health and wellbeing, and devolved powers for Scotland	(Citizen’s Assembly Scotland, 2020, Scottish Government, 2021, Elstub et al., 2022)

**Table 4:** Differing narratives

Recommendation no. 5	Recommendation no. 10
People don’t know how to care for themselves when they leave school as they are not taught any life skills. This impacts local people as they don’t know how to apply for a job, [make] a decent meal or pay a bill. This results in lots of unemployment in Blackpool and people with health and weight problems as they don’t know how to create something tasty yet healthy. This could be fixed by teaching life skills in schools so when the students leave they can lead a healthy and happy lifestyle.	Employers need to provide a living wage and work contracts that give you security and employee benefits (sick pay, holiday pay, pension) and not just seasonal or temporary contracts. The impact of this is unemployment, poverty, lack of life quality, health problems, lack of security, inequality and crime. This has come about because of austerity, lack of investment, the erosion of workers rights, a target driven culture, privatization and a failure to value workers
