# Supplementary material for: Participatory-deliberative processes in UK policymaking related to income insecurity as a determinant of health: a scoping review
Source: Evid Policy. Author manuscript; Available in PMC 2025 May 14. (PMC7617669; doi:10.1332/17442648Y2025D000000053)
Supplement: Supplementary File [file EMS205272-supplement-Supplementary_File.pdf]

Evidence and Policy article, March 2025:

Participatory-deliberative processes in policymaking related to income insecurity as a determinant of health: a scoping review

Supplementary File: Search Strategy (completed 2022)

EconLit Searches (EBSCO)

Studies excluded as NON-UK / NON-DI / NON-INCOME = **1,186**

Studies put forward for SCOPING Review STAGE 1 SELECTION: 61 (no duplicates)

S7	**S5 AND S6 limited to 2007 onwards**	**1,353**
S6	TI (((poor or lived-experience or public or citizen* or communit* or lay or household* or low-income or stakeholder*) N3 (participat* or deliberat* or “decision-making” or community or democra* or involve* or partners* or citizen* or panel or assembl* or neighbourhood* or jury or juries or collabor* or forum or committee or co-governance))) OR AB (((poor or lived-experience or public or citizen* or communit* or lay or household* or low-income or stakeholder*) N3 (participat* or deliberat* or “decision-making” or community or democra* or involve* or partners* or citizen* or panel or assembl* or neighbourhood* or or jury or juries or collabor* or forum or committee or co-governance)))	17,252
S5	**S1 OR S2 OR S3 OR S4**	67,034
S4	TI (Policy N3 (social or making or public or economic or decision making socio-economic or inequalit*)) OR AB (Policy N3 (social or making or public or economic or decision making or socio-economic or inequalit*))	52,731
S3	TI (Democr* N3 (economic or innovate* or deliberat*) OR AB (Democr* N3 (economic or innovate* or deliberat*)	1,656
S2	TI (Insecurity N3 (financial or economic or job or income) OR AB (Insecurity N3 (financial or economic or job or income)	804

S1	TI (Income N3 (insecurity or poverty or depriv* or security or inequalit* or austerity)) OR AB (Income N3 (insecurity or poverty or depriv* or security or inequality* or austerity))	13,601

<u>SOC Index Searches</u> (EBSCO)

Non-UK /Non-DI / Non-Income = 1,442

Studies put forward for SCOPING Review STAGE 1 SELECTION: 89 (duplicates x 2 = 87)

S7	**S5 AND S6 limited to 2007 onwards**	**1,532**
S6	TI (((poor or lived-experience or public or citizen* or communit* or lay or household* or low-income or stakeholder*) N3 (participat* or deliberat* or “decision-making” or community or involve* or partners* or citizen* or panel or assembl* or neighbourhood* or jury or juries or collabor* or forum or committee or co-governance))) OR AB (((poor or lived-experience or public or citizen* or communit* or lay or household* or low-income or stakeholder*) N3 (participat* or deliberat* or “decision-making” or community or involve* or partners* or citizen* or panel or assembl* or neighbourhood* or or jury or juries or collabor* or forum or committee or co-governance)))	45,357
S5	**S1 OR S2 OR S3 OR S4**	68,674
S4	TI (Policy N3 (social or making or public or economic or decision making socio-economic or inequalit*)) OR AB (Policy N3 (social or making or public or economic or decision making or socio-economic or inequalit*))	58,748
S3	TI (Democr* N3 (economic or innovate* or deliberat*) OR AB (Democr* N3 (economic or innovate* or deliberat*)	2,654
S2	TI (Insecurity N3 (financial or economic or job or income) OR AB (Insecurity N3 (financial or economic or job or income)	1,409
S1	TI (Income N3 (insecurity or poverty or depriv* or security or inequalit* or	6,981

	austerity)) OR AB (Income N3 (insecurity or poverty or depriv* or security or inequality* or austerity))	

<u>MedLine Searches</u> (EBSCO)

Non-UK /Non-DI / Non-Income = 2,285

Studies put forward for SCOPING Review STAGE 1 SELECTION: 89 (no duplicates)

S7	**S5 AND S6 limited to 2007 onwards**	**2,580**
S6	TI (((poor or lived-experience or public or citizen* or communit* or lay or household* or low-income or stakeholder*) N3 (participat* or deliberat* or “decision-making” or community or democra* or involve* or partners* or citizen* or panel or assembl* or neighbourhood* or jury or juries or collabor* or forum or committee or co-governance))) OR AB (((poor or lived-experience or public or citizen* or communit* or lay or household* or low-income or stakeholder*) N3 (participat* or deliberat* or “decision-making” or community or democra* or involve* or partners* or citizen* or panel or assembl* or neighbourhood* or or jury or juries or collabor* or forum or committee or co-governance)))	83,039
S5	**S1 OR S2 OR S3 OR S4**	50,282
S4	TI (Policy N3 (social or making or public or economic or decision making socio-economic or inequalit*)) OR AB (Policy N3 (social or making or public or economic or decision making or socio-economic or inequalit*))	43,228
S3	TI (Democr* N3 (economic or innovate* or deliberat*) OR AB (Democr* N3 (economic or innovate* or deliberat*)	259
S2	TI (Insecurity N3 (financial or economic or job or income) OR AB (Insecurity N3 (financial or economic or job or income)	1,975
S1	TI (Income N3 (insecurity or poverty or depriv* or security or inequalit* or austerity)) OR AB (Income N3	5,567

	(insecurity or poverty or depriv* or security or inequality* or austerity))	

Sociological Abstract Searches (PROQUEST)

Non-UK /Non-DI / Non-Income = 2,776

Studies put forward for SCOPING Review STAGE 1 SELECTION: 250 (no duplicates)

S5	**Limit English and UK**	**3,035**
S4	Limit to 2007 onwards	140,195
S3	S1 AND S2	261,656
S2	ab (poor or lived-experience or public or citizen* or communit* or lay or household* or low-income or stakeholder* or participat* or deliberat* or “decision-making” or community or democra* or involve* or partners* or citizen* or panel or assembl* or neighbourhood* or jury or juries or collabor* or forum or committee or co-governance) or ti (poor or lived-experience or public or citizen* or communit* or lay or household* or low-income or stakeholder* or participat* or deliberat* or “decision-making” or community or democra* or involve* or partners* or citizen* or panel or assembl* or neighbourhood* or jury or juries or collabor* or forum or committee or co-governance)	676,898
S1	ab("income insecurity" OR poverty OR depriv* OR security OR inequalit* OR austerity or "financial insecurity" or "economic insecurity" or "job insecurity" or Democr* or "economic democracy" or "democratic innovations" or "deliberative democracy" or or "participatory democracy" or policy* or "social policy" or "public policy" or economic or "decision making" or socio-economic or inequalit*) OR ti("income insecurity" OR poverty OR depriv* OR security OR inequalit* OR austerity or "financial insecurity" or "economic insecurity" or "job insecurity"	445,475

	or Democr* or "economic democracy" or "democratic innovations" or "deliberative democracy" or or "participatory democracy" or policy* or "social policy" or "public policy" or economic or "decision making" or socio-economic or inequalit*)	

Grey Literature

BASE Database Search **TOTAL RESULTS: 1,772**

Key terms search (limited to UK & 2007 onwards)

- Democratic innovation – 945; **references put forward: 70**
- Income Insecurity – 56; **references put forward: 0 (all related to food insecurity)**
- Participation – 731; **references put forward: 39**
- Deliberative – 40; **references put forward: 4**

Other search terms:

poverty / socioeconomic policy / decision-making / lived experience

Checked the first 100 results of each search and most resulted in:

- largely irrelevant studies
- too many studies to go through (+ 20,000), with the first 100 not meeting criteria

In total docs put forward for next selection: 107

Websites

- **Scottish Government** **TOTAL RESULTS: 2,458**

Initial Search:

- Publications
- Topics:
  - Communities / third sector – **Results: 1651 (all docs)**

    In total docs put forward for next selection: 17

Next searches:

- **Economy**
- **Constitution/ Democracy**
- **Money and tax**

Limited to consultation analysis/ report, strategy /plan, impact assessment, progress report, independent report, research and analysis**: 798,** **put forward: 5**

- **Social Security Scotland**
  - Lived experience panels: 9 results, **1 put forward**

- **UK Government website proper** **TOTAL RESULTS: 966**

Searches in Research and Statistics and Policy Papers and Consultations

Key word:

- **Participation** in:
  - Welfare – results **72**, **put forward 2**
  - Government – results **319**, **put forward 3**
  - Money – results **81**, **1 put forward**

Topic search:

- **Poverty and social justice**, policy and consultations: **13** results, **1 put forward**
- **Community and society,** policy and consultations: **444** results, **3 put forward**
- **Social Action,** policy and consultations: **37** results, **0 put forward**

- **JRF** **TOTAL RESULTS: 422**

Key word searches:

Participation, 2007 onwards: 195

Decision-making, income and benefits, 2007 onwards: 227

Studies put forward: 10

- **IPPR** **TOTAL RESULTS: 315**

Key word searches:

Participation – 156 results, **3 put forward**

Citizens – 109 results, **3 put forward**

Democratic innovation, economy – 34 results, **3 put forward**

Citizen's Jury, economy – 16 results, **1 put forward**

- **Participedia.org** **TOTAL RESULTS: 263**

Limited to UK CASE STUDIES – 263, **17 put forward**

Question mark over the inclusion of participatory budgeting as separate case studies

- **Involve** **TOTAL RESULTS: 81**

All publications and projects: **2 put forward**

- **COSLA** **TOTAL RESULTS: 320**

Key words:

- Participation

Topic area: **communities**, results **40**, **put forward 2**

Topic area, **local government finance,** results **20, put forward 0**

Topic area, **environment and economy**, results **260**, **put forward 2**

- **Local Government Association** **TOTAL RESULTS: 327**

Key words in publications:

- Participation: results **49**, **put forward 3**

Publication by topic area:

- Devolution, economic growth, business and finance rates**:** results **65, put forward 1**
- Welfare reform, communities**:** results **35, put forward 0**

Case studies by topic area**:**

- Devolution, economic growth, business and finance rates, communities, welfare reform: results **178, 0 put forward**
